# A serial mediation model of social media addiction and college students’ academic engagement: the role of sleep quality and fatigue

**DOI:** 10.1186/s12888-023-04799-5

**Published:** 2023-05-12

**Authors:** Jie Zhuang, Qiaoxing Mou, Tong Zheng, Fei Gao, Yaqin Zhong, Qingyun Lu, Yuexia Gao, Miaomiao Zhao

**Affiliations:** 1 Department of Health Management, School of Public Health, Nantong University, Nantong, Jiangsu China; 2 Department of medical administration, The First Psychiatric Hospital of Harbin, Harbin, Heilongjiang China; 3 Center for Food Safety and School Health, Heilongjiang Provincial Center for Disease Control and Prevention, Harbin, Heilongjiang China

**Keywords:** Social media addiction, Academic engagement, Sleep quality, Fatigue, Serial mediation model

## Abstract

**Background:**

It has been documented that social media addiction (SMA) has a detrimental effect on college students’ academic engagement. However, the mechanisms underlying this association are not well understood. This study aimed to determine the serial mediation effects of sleep quality and fatigue on the relationship between SMA and academic engagement among college students.

**Methods:**

A cross-sectional survey was conducted with 2661 college students (43.3% males, mean age = 19.97 years). The participants completed the Bergen Social Media Addiction Scale, the Utrecht Student Work Engagement Scale for Students, the Pittsburgh Sleep Quality Index, and the Fatigue Assessment Scale. The serial mediation effects were examined using Model 6 in the Hayes’ PROCESS macro for SPSS.

**Results:**

The results showed that SMA among college students had a direct negative relationship with their academic engagement (Effect = − 0.051, 95% CI: −0.087 to − 0.015). In addition, sleep quality and fatigue mediated the relationship between SMA and academic engagement both independent and serially, with the independent mediation effect of sleep quality being − 0.031 (95% CI: −0.048 to − 0.016), the independent mediation effect of fatigue being − 0.109 (95% CI: −0.133 to − 0.088), and the serial mediation effect of sleep quality and fatigue being − 0.080 (95% CI: −0.095 to − 0.066). The total indirect effect of the three mediation paths was 80.9%.

**Conclusions:**

Decreased academic engagement caused by SMA can be aggravated by poor sleep quality and fatigue. Strengthening supervision and intervention in social media use among college students, supplemented by attention to psychosomatic health, including sleep quality and fatigue could promote their engagement in academic work.

## Background

In this era of information explosion, people exchange information and interact with entertainment through social media networks. According to the China Internet Network Information Center, the number of Internet users in China was 1.067 billion by December 2022, with an increase of 35.49 million from December 2021, and the Internet penetration rate had reached 75.6% [1]. Social media comprises Internet-based content production and exchange platforms built on Web 2.0 for users to communicate and share information [2]. Currently, China’s domestic social media platforms include WeChat, QQ, and short video-sharing platforms such as TikTok and Kwai. With the development of WEB2.0, the number of loyal social media users had rapidly expanded. According to Global Social Media User Statistics, China had 1.021 billion social media users as of 2023, ranking first worldwide [3]. Teenagers and young adults continue to dominate the China’s social media users base [4].

Although the wide variety of social media applications and the extremely high popularity of social media among the younger generations afford conveniences and fresh user experiences to those active in online social networking, addiction to social media is a public health concerns that must be faced squarely [5]. Social media addiction (SMA) is defined as an excessive concern about social networking, a burning drive to consume it, and a commitment of so much time and attention to social media that it impairs other crucial aspects of life such as social activities, interpersonal relationships, studies/work, and health and well-being [6]. SMA may even lead to pathological psychological dependence on social media with symptoms of behavioral addiction [7]. This uncontrollable overuse of social media can be explained by the general model of addiction and is considered a component of Internet addiction [8, 9].

SMA has been reported to have negative effects on students’ learning, such as distraction, severe procrastination, and decreased academic productivity [10, 11]. However, few studies have examined the mechanisms underlying the effects of SMA on academic engagement among college students. Particularly, psychosomatic disorders including decreased sleep quality and fatigue, the adverse outcomes of SMA, have rarely been examined as important links in the possible internal mechanisms of this influence. This information can indicate effective measures to prevent and reduce the consequences of SMA among college students and improve the quality of learning for those with addiction.

### SMA and academic engagement

Academic engagement is a positive and fulfilling mental state related to learning, that is embodied in the planning and practical actions of students in the learning process and is characterized by vitality, dedication and concentration [12]. Academic engagement represents the time students devote to academic pursuits and the quality of their educational endeavors [13]. It can serve as a proxy for students’ learning quality [14] and is closely connected with academic achievement and personal success [15, 16].

Studies have shown that SMA negatively affects students’ academic engagement [17]. Internet addiction theory suggests that individuals who are addicted to the Internet gradually lose self-control over time [18]. The addictive use of social media frequently involves spending more time connecting to social media tasks for entertainments than studies. However, students often underestimate the appeal of social media while overestimating their self-control, are easily distracted by the entertainment and social features offered by social media, and fail to concentrate on learning [17]. According to the Strength Model of Self-Control (SMSC), individual’s cognitive and psychological resources are limited, and the resources expend on some tasks are bound to discourage resources expend on others [19]. The vitality, dedication and concentration contained in academic engagement are important psychological resources that individuals need to complete learning tasks. However, excessive social media involvement requires a large amount of concentration and vitality that consumes individual’s resources, thus leading to insufficient resources devoted to academic activities. Consequently, students addicted to social media may become overwhelmed when face with learning tasks that requiring absolute focus and cognitive ability.

#### The mediating role of sleep quality

Sleep is not only necessary for biological functions but is also an important part of life. Sleep quality is affected by various biological, psychosocial, and environmental factors.

Numerous studies have reported a strong association between social network sites use and poorer sleep quality among young adults [20–22]. Several mechanisms may explain the adverse effects of social media use on sleep quality. First, the time spent on social media may replace that spent sleeping, leading to delayed bedtimes and shortened sleep duration [23]. Second, improper timing of exposure to the bright light of electronic devices can disrupt circadian rhythms, resulting in insomnia [24]. Third, electronic device use may cause more difficulties falling asleep by increasing cognitive and affective arousal at bedtime, which may, in turn, lead to more difficulty falling asleep and poorer sleep quality [25].

Sleep quality is an important factor affecting academic engagement. Decreased sleep quality impairs cognitive function [26], leading to decreases in memory capacity and logical thinking skills [27, 28], which are associated with students’ academic engagement. In a meta-analysis, Dewald et al. indicated that sleep quality and duration had the greatest impact on students’ learning [29]. In addition, sleep deprivation causes impairs memory performance and reduced academic effort [30], and some students may experience subjective slacking due to poor sleep quality, leading to reduced academic motivation [31]. Previous study has problematic social media use may have a negative effect on academic through disturbed sleep, which is supported by previous studies [32].

### The mediating role of fatigue

Fatigue is a subjective feeling of tiredness or persistent physical exhaustion, which includes the experience of fatigue and its effects on the physical, psychological, and social aspects of life [33]. Fatigue is often considered a multidimensional construct with various aspects, such as physical and mental fatigue [34]. Fatigue is common among college students [35] among whom it is associated with behavioral, cognitive, and affective factors [36]. One behavioral factor that deserves attention is Internet addiction. A study conducted among Moroccan university students found higher levels of mental and physical fatigue in students with Internet addiction than in those without Internet addiction [37]. As a subtype of Internet addiction, SMA can have similar consequences. Addictive students inevitably access to social media during class, which leads to multitasking. As Stanley noted, addicts tend to constantly check social media and change from one task to another on social media; such information overload leads to fatigue and exhaustion [38].

Fatigue has negative consequences for students’ learning. Some studies have shown that subjective chronic fatigue, as a psychophysiological factor, is one of the main reasons for school avoidance [39]. Students who felt fatigued tended to show worse learning motivation [40], that is, subjective exhaustion greatly affected their enthusiasm for devoting their energy to academic activities. A decrease in enjoyment and motivation for attending school can lead to significant absenteeism or an impertinent attitude toward learning that compromises academic engagement [41]. Thus, those who are addicted to social media may experience mental laziness and delays in action, leading to poor academic engagement.

### The serial mediating role of sleep quality and fatigue

The relationship between sleep and fatigue has been explored previously [42]. One cause of fatigue is the disruption of circadian rhythms caused by sleep deprivation. Fatigue is due to sleep deprivation [43], and insomnia is associated with significantly higher levels of fatigue than in those who are not diagnosed with insomnia [44]. Prospective daily diary studies also found that subjective sleep quality plays a key role in predicting self-reported daytime fatigue [45, 46]. These findings support the notion that sleep quality are antecedents of fatigue.

From the viewpoint of energy-depleting process of Developmental-Contextual Model of Digital Demands and Resources (DC-DDR), negative outcomes from digital media result from the imbalance between demands and resources available to overcome these demands [47]. The energy-depleting process occur if digital activities take too much time or effort, simultaneously displacing other important daily activities. Based on this theory, poor sleep quality due to excessive use of social media will cause psychological demands and resources consumptions, further forming an imbalance between demands and resources, leading to increased demands and feelings of exhaustion the following day, and in turn resulting in a decrease in fatigue-induced academic engagement [47, 48].These findings and theories provide evidence linking the SMA to academic engagement sequentially through sleep quality and fatigue.

### The present study

In summary, this study aim to test a serial mediation model linking SMA to academic engagement among college students through the psychosomatic health factors of sleep quality and fatigue. Drawing on SMSC theory, tasks processing requires mental and cognitive resources, and people have only a limited pool of resources. Multitasking collectively consumes resources and create competition for them. Thus, addicted students who are heavily immersed in social media activities will inevitably interfere with and damage learning activities. Literature analyses have supported the note that sleep quality and fatigue, as psychosomatic health factors, are the negative consequences of SMA, and have also been linked to academic problems. According to the DC-DDR theory, the energy-depleting process occur when addictive use of social media interferes with sleep quality, which further forming an imbalance between demands and resources that increase next demands and resources consumptions, thus lead to fatigue and eventually disrupt academic engagement. Grounded in the aforementioned theories and findings, we propose the following hypotheses:

***H1.*** *SMA is negatively associated with academic engagement.*

***H2.*** *Sleep quality mediates the relationship between SMA and academic engagement.*

***H3.*** *Fatigue mediates the relationship between SMA and academic engagement.*

***H4.*** *Sleep quality and fatigue play a serial mediating role in the relationship between SMA and academic engagement.*

## Methods

### Participants

The data used in this study were extracted from a larger cross-sectional study of mental, and behavioral factors related to health and academics among college students, conducted using a convenience sampling method from November 2020 to January 2021 at a university in Nantong, Jiangsu, China. The sample size was determined according to Hair’s recommendation that the ratio of observations to variables of 20:1 [49], and was increased by 10% to overcome non-response. The desired sample size was thus 2,112. As this survey was conducted in a university school, we obtained the verbal informed consent from the students’ university counselor teachers, who are the guardians of the students before the survey. All the participants received thorough explanations of the detailed instructions on the purpose, procedure, and the voluntary and confidentiality nature of this study; and provided informed consent. A total of 2,947 students participated in this survey. After data cleaning, 286 questionnaires with the omission of key study variables or logical errors were excluded, resulting in the inclusion of 2,661 participants in the present study (efficiency rate of 90.3%).

### Measures

#### Basic characteristics

The basic characteristics of the participants including gender, age, academic major, and average daily social media use, were collected.

#### The bergen social media addiction scale

The Bergen Social Media Addiction Scale (BSMAS) was used to evaluate SMA [50]. The BSMAS is based on The Bergen Facebook Addiction Scale, with expanding the term “Facebook” to the broader context of “social media” and gives specific applications of social media, such as “Facebook, Twitter, Instagram, etc.” .The BSMAS reflects the core elements of addiction in six items, with each item scored on a 5-point Likert scale ranging from 1 (*very rarely*) to 5 (*very often*), with a higher score reflecting a deeper degree of SMA. Individuals with a total score equal to or greater than 19 were considered to have SMA [51]. In this study, the Cronbach’s α of the BSMAS was 0.877.

#### The utrecht work engagement scale-student

The Utrecht Work Engagement Scale-student (UWES-9 S) was used to measure academic engagement [52]. The UWES-9 S was developed on the basis of the Utrecht Work Engagement Scale and consists of nine items divided into three components: vigor, dedication, and absorption. Each item is scored on a 7-point Likert scale ranging from 0 (*never*) to 6 (*always*), with higher scores indicating higher academic engagement. The Chinese version of the UWES-9 S has demonstrated good reliability and validity [53]. In this study, Cronbach’s α of the UWES-9 S was 0.959.

#### The pittsburgh sleep quality index

The Pittsburgh Sleep Quality Index (PSQI) is a self-rated questionnaire that assesses the sleep quality of subjects over the past month [54]. The PSQI consists of 18 self-evaluation items classified into seven components including subjective sleep quality, sleep duration, sleep duration, sleep latency, sleep disturbances, habitual sleep efficiency, sleeping medication use, and daytime dysfunction. Each component is scored on a 4-point scale ranging from 0 to 3. The total score on the scale is the sum of the scores for these seven components, resulting in a total score ranging from 0 to 21. A higher total PSQI score indicated a poorer sleep quality. In this study, the Cronbach’s α of the PSQI was 0.691.

#### The fatigue assessment scale

The Fatigue Assessment Scale (FAS) was used to measure fatigue [55]. The FAS is composed of ten items and each item is scored on a 5-point Likert scale ranging from 1 (*never*) to 5 (*always*), with higher scores indicating higher levels of subjective fatigue. This scale has been widely used as an effective self-reporting instrument measuring subjective fatigue [56, 57]. In this study, Cronbach’s α of the FAS was 0.758.

### Statistical analyses

Data analyses were performed using IBM SPSS version 25.0 (IBM SPSS Statistics for Windows, IBM Corp., Armonk, NY, United States). Descriptive statistics were used to present the participants’ demographic characteristics. Pearson correlation coefficients were used to examine the correlations between the study variables. The PROCESS macro for SPSS (Model 6) was then used to test the serial mediation effect. A bootstrap test with 5,000 repeat samplings was used to test the statistical robustness, with a 95% confidence interval (CI) not containing 0 indicating a significant moderating effect [58]. Students’ gender, age, and academic major were controlled in the models. The Harman single-factor test [59] was performed to test for the common method bias, with the total variance extracted by the first factor below 50% representing the absence of common method bias [60]. The variance inflation factor (VIF) was performed to test for the multicollinearity of the study variables, with VIF value of each variable below 5 representing a low concern for multicollinearity [61].

## Results

### Basic characteristics

Of the 2,661 participants, 1,153 (43.3%) were male and 1,508 (56.7%) were female, with ages ranging from 16 to 24 years (19.97 ± 1.253). The numbers of participants with medical and non-medical majors were 995 (37.4%) and 1,666 (62.6%), respectively. The average daily time that the participants spent on using social media was 3.02 ± 2.42 h. According to the BSMAS, 4.1% of the participants were found to have SMA.

### Testing for common method bias

A total of 10 factors with eigenvalues greater than 1 were identified with the first factor having an explanatory rate of 28.97%, which was below the recommended threshold of 50%. This indicated no serious common method bias in the data.

### Testing for multi-collinearity

The collinearity diagnostics showed that the VIFs of the study variables were below the recommended threshold of 5, demonstrating a low concern for multicollinearity in the data.

### Descriptive statistics and correlation analysis

Table 1 presents the means, standard deviations (SD), and Pearson’s correlation coefficients for SMA, sleep quality, fatigue, and academic engagement. The results demonstrated a significant correlation between the study variables. SMA was significantly positively correlated with poor sleep quality (*r* = 0.357, *p* < 0.001; higher PSQI scores indicating poor sleep quality) and fatigue (*r* = 0.417, *p* < 0.001), whereas it was significantly negatively correlated with academic engagement (*r* = − 0.270, *p* < 0.001). Poor sleep quality was significantly positively correlated with fatigue (*r* = 0.589, *p* < 0.001) and significantly negatively correlated with academic engagement (*r* = − 0.371, *p* < 0.001). Fatigue was significantly and negatively correlated with academic engagement (*r* = − 0.523, *p* < 0.001). The significant correlations among the study variables provide initial support for the proposed hypotheses.

**Table 1:** Descriptive statistics and Pearson correlations of the study variables

Variable	1	2	3	4	5	6	7
1. SMA	1						
2. Sleep quality	0.357^***^	1					
3. Fatigue	0.417^***^	0.589^***^	1				
4. Academic engagement	−0.270^***^	−0.371^***^	−0.523^***^	1			
5. Gender	−0.049^*^	0.069^**^	0.108^*^	−0.062^**^	1		
6. Age	−0.043^*^	0.064^**^	0.001	0.004	0.167^**^	1	
7. Academic major	−0.071^**^	0.110^**^	0.049^*^	−0.050^*^	0.014	0.047^*^	1
*M*	10.03	5.02	21.57	30.30	—	19.97	—
*SD*	4.509	3.052	6.376	10.499	—	1.253	—

Note. *SMA* = Social media addiction; *M* = mean; *SD* = standard deviation. Gender is a dummy code (male = 1; female = 0). Academic major is a dummy code (medical = 1; non-medical = 0). ^*^p < 0.05, ^**^p < 0.01, ^***^p < 0.001

### Testing the serial mediation effect

**Fig. 1:**
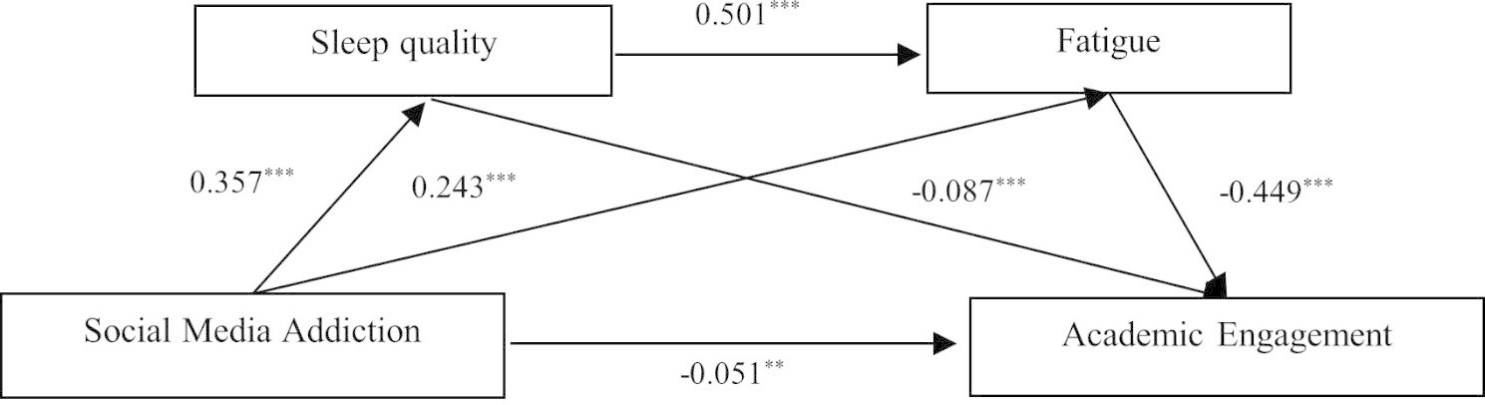
The serial mediation effect

Model 6 in the SPSS PROCESS macro was used to test the serial mediation effect. The results of the regression analysis (Table 2) show that SMA was significantly positively associated with poor sleep quality (*β* = 0.357, *p* < 0.001), which in turn was significantly negatively associated with academic engagement (*β* = − 0.087, *p* < 0.001); meanwhile, the direct effect of SMA on academic engagement was significant (= − 0.051, *p *< 0.01); supporting Hypotheses H1 and H2. Moreover, SMA was significantly positively associated with fatigue (*β* = 0.243, *p* < 0.001), which in turn was significantly negatively associated with academic engagement (*β* = − 0.449, *p* < 0.001), supporting Hypothesis H3. Finally, poor sleep quality was significantly positively associated with fatigue (*β* = 0.501, *p* < 0.001), forming a serial mediation pathway that supported Hypothesis H4. The detailed path model was shown in Fig. 1.

**Table 2:** Regression analysis of variable relationship in serial mediation model

Result variable	Predictor variable	*R*	*R²*	*F*	*β*	*t*
Sleep quality	SMA	0.382	0.146	113.114^***^	0.357	19.838^***^
	Gender				0.150	4.082^***^
	Age				0.051	3.485^***^
	Academic major				0.167	4.477^***^
Fatigue	SMA	0.637	0.405	361.853^***^	0.243	15.061^***^
	Sleep quality				0.501	30.960^***^
	Gender				0.185	5.889^***^
	Age				−0.028	−2.333^*^
	Academic major				−0.048	−1.539
Academic engagement	SMA	0.531	0.282	174.052^***^	−0.051	−2.781^**^
	Sleep quality				−0.087	−4.175^***^
	Fatigue				−0.449	−21.050^***^
	Gender				−0.023	−0.671
	Age				0.008	0.637
	Academic major				−0.031	−0.911

Note. SMA = Social media addiction. Gender, age, and academic major were controlled in the model. The study variables, excluding the control variables were standardized in the model. ^*^p < 0.05, ^**^p < 0.01, ^***^p < 0.001

The bootstrap test results (Table 3) showed that sleep quality and fatigue partially mediated the relationship between SMA and academic engagement, with a total indirect effect of − 0.220, accounting for 80.9% of the total effect. Specifically, the mediating effect was composed of indirect effects generated by three pathways: (1) SMA → sleep quality → academic engagement (effect = − 0.031, 95% CI: −0.048 to − 0.016), accounting for 11.4% of the total effect; (2) SMA → fatigue → academic engagement (effect = − 0.109, 95% CI: −0.133 to − 0.088), accounting for 40.1% of the total effect; and (3) SMA → sleep quality → fatigue → academic engagement (effect = − 0.080, 95% CI: −0.095 to − 0.066), accounting for 29.4% of the total effect.

**Table 3:** The total, direct and indirect effect of social media addiction on academic engagement

	Effects	Boot SE	Boot LLCI	Boot ULCI
Total effect	−0.272	0.019	−0.308	−0.235
Direct effect	−0.051	0.018	−0.087	−0.015
Total indirect effect	−0.220	0.017	−0.255	−0.187
Indirect effect 1	−0.031	0.008	−0.048	−0.016
Indirect effect 2	−0.109	0.011	−0.133	−0.088
Indirect effect 3	−0.080	0.007	−0.095	−0.066

Note: Boot SE, Boot LLCI, and Boot ULCI refer to the standard error and the upper and lower bounds of the 95% confidence intervals of the indirect effects estimated by the bootstrap method, respectively. Indirect effect 1: social media addiction → sleep quality → academic engagement; indirect effect 2: social media addiction →fatigue → academic engagement; indirect effect 3: social media addiction → sleep quality →fatigue → academic engagement

## Discussion

Academic engagement, recognized as an important influence on achievement and learning, has garnered significant research [62, 63]. In the current information age, social media is gradually penetrating all aspects of life, and the impact of social media use on college academic engagement has been revealed [64]. However, the underlying mechanism remains largely unexplored. This study examined the relationship between SMA and academic engagement in college students, with a specific focus on the serial mediating effect of sleep quality and fatigue, contributing to the existing literature by expanding our understanding of the negative effects of addictive behaviors on academic issues among college students through the pathway of deteriorating psychosomatic health. Our findings showed that SMA was significantly negatively associated with academic engagement, and sleep quality and fatigue functioned as separate and serial mediators in the relationship between SMA and academic engagement.

### SMA and academic engagement

Confirming Hypothesis H1, we found a directly negative relationship between SMA and academic engagement, suggesting that SMA is an important risk factor for low academic engagement. This finding corroborates previous studies [17]. Research has confirmed that social media imposes an auditory and visual burden that makes it difficult for students to use the same amount of energy to deal with the impact of information from social media and the large amount of academic information that must be processed simultaneously, thereby reducing academic engagement [10], in line with the SMSC theory. In addition, flow theory suggests that individuals who are immersed in the task performed will lose control of the time spent on this task, and their attention to any other demand/task is disregarded [65]. In this process, one will experience enjoyment in interacting with the task and feels in control of it, and any outside interference is unimportant. The flow state can be achieved in a social media environment when a person is fully immersed in an activity [66]. Hence, students addicted to social media typically immerse themselves in the online world and fade out of learning activities in the offline world.

#### The mediating role of sleep quality

This study found that sleep quality mediated the relationship between SMA and academic engagement, supporting Hypothesis H2. Compensatory Internet use theory proposes that an individual’s overuse of social media is a mechanism to compensate for negative feelings or psychopathy [67]. Self-determination theory postulates that individuals may self-regulate their behavior to fulfill their intrinsic and extrinsic needs [68]. Individuals may regulate their sleep patterns and sleep time to stay on social media that meets their psychological needs, thereby contributing to sleep problems.

The negative effect of poor sleep quality on student learning can be explained by the function of sleep on cognition and memory. Poor sleep quality impairs attention and affects cognitive functions governed by the brain, including language learning and abstract logical thinking, which are extremely important for learning inputs [69]. The brain region that is most significantly affected by low-quality sleep is the prefrontal cortex, which performs higher-level brain functions, including concentration, working memory, and logical reasoning. Similarly, sleep deprivation has been shown to reduce memory encoding, suggesting that the hippocampus is affected, leading to reduced knowledge retention [70]. Therefore, students with poor sleep quality may have more difficulty grasping classroom materials, ultimately reducing their academic engagement [71].

#### The mediating role of fatigue

Supporting of H3, we found that fatigue mediates the relationship between SMA and academic engagement. In the Internet age, the Internet and social media are considered stimulus that can cause several negative outcomes [72]. The extensive use of social media has exposed people to massive amounts of information and communication demands that require considerable energy and cognitive processing, resulting in cognitive overload [73]. Thereby, students who are addicted to exciting and interesting online social activities constantly encounter a significantly higher neurological load than those who are not addicted, and they are more likely to experience fatigue.In addition, persistent mental fatigue has been shown to lead to increased distractibility, and poor information processing [74, 75], which are related with reduced academic attainment in college students [76]. Therefore, excessive brain activity and stimulation can cause mental fatigue, akin to physical fatigue, making uncomplicated tasks increasingly difficult or even impossible [77]. This could explain our hypothesis that, as students’ fatigue increases owing SMA, their academic engagement may be negatively affected.

#### The serial mediating role of sleep quality and fatigue

The key finding of this study was that the association between SMA and academic engagement was partially mediated by sleep quality and fatigue through a sequential pathway, supporting Hypothesis H4. However, the underlying mechanism was not well explored in previous studies, few of which have explored the role of sleep quality and fatigue in the relationship between social media use and learning among college students. The interrelated physiological mechanisms of sleep and fatigue could provide explains. An association between sleep quality and mental fatigue has been demonstrated for the neuroendocrine pathways. As a neuroendocrine factor, cortisol, which is elevated under the influence of sleep disorders, can lead to abnormal activity in the hypothalamic-pituitary-adrenal cortex, negatively affecting mental fatigue and daytime excitability [78]. Nocturnal sleep disturbances affect daytime functioning, which is crucial for students’ performance during waking hours, and sleep plays a key role in cognitive function [79]. Thus, students who are addicted to social media experience daytime sleepiness, poor concentration, reduced cognitive performance, and a tendency to devote less effort to their tasks [80], making problems with academic engagement unavoidable. Therefore, the serial mediation model in this study provides new insights into the psychosomatic consequences of addiction to social media, with poor sleep quality and fatigue mediating the negative effect of SMA on academic engagement.

### Limitations

This study had some limitations. First, because this study had a cross-sectional design, it was not possible to establish a causal relationship between the variables. In future work, the causal relationship between SMA and academic engagement could be explored through longitudinal follow-ups. Second, due to the limitations of research funds and the time, this study only conducted a questionnaire survey at one university in Nantong; therefore, whether our findings are generalizable needs to be treated with caution. Finally, the data used in this study were obtained through self-administered questionnaires by students, which might have caused some subjective bias.

## Conclusion

The present study provides empirical evidence for the serial mediating roles of sleep quality and fatigue in the relationship between SMA and academic engagement, which contributes to existing knowledge on the underlying mechanism of addictive behaviors in academic engagement from a psychosomatic perspective. This study found that the decreased academic engagement caused by SMA could be aggravated by poor sleep quality and fatigue. Therefore, interventions should consider way to encourage college students who experience SMA to improve sleep quality and reduce fatigue to promote their academic engagement. Even if we do not use these results in interventions to solve the addiction, at least we can reduce the negative effects of social media on students’ lives by providing recommendations to students who are addicted to it.

## Data Availability

The datasets used during the current study are available from the corresponding author on reasonable request.

## Abbreviations

SMA: Social media addiction
DC-DDR: Developmental-Contextual Model of Digital Demands and Resources
SMSC: the Strength Model of Self-Control
BSMAS: Bergen Social Media Addiction Scale
UWES-9S: Utrecht Work Engagement Scale-student
PSQI: Pittsburgh Sleep Quality Index
FAS: Fatigue Assessment Scale
CI: confidence interval
VIF: variance inflation factor
SD: standard deviations
